# Fabrication and In Vitro Characterization of Electrochemically Compacted Collagen/Sulfated Xylorhamnoglycuronan Matrix for Wound Healing Applications

**DOI:** 10.3390/polym10040415

**Published:** 2018-04-09

**Authors:** Lingzhi Kang, Xiao Liu, Zhilian Yue, Zhi Chen, Chris Baker, Pia C. Winberg, Gordon G. Wallace

**Affiliations:** 1 ARC Centre of Excellence for Electromaterials Science, Intelligent Polymer Research Institute, Innovation Campus, University of Wollongong, Wollongong, NSW 2522, Australia; lk538@uowmail.edu.au (L.K.); xiaol@uow.edu.au (X.L.); zc555@uowmail.edu.au (Z.C.); 2 Department of Dermatology, St Vincent’s Hospital Melbourne, Melbourne, VIC 3065, Australia; chris.baker@svha.org.au; 3 Department of Medicine (Dermatology), University of Melbourne, Melbourne, VIC 3010, Australia; 4 Venus Shell Systems Pty Ltd., 220 Bolong Road, Bomaderry, NSW 2541, Australia; pia@venusshellsystems.com.au or pia@uow.edu.au; 5 School of Medicine, SMAH, University of Wollongong, Wollongong, NSW 2500, Australia

**Keywords:** sulfated xylorhamnoglycuronan, electrocompaction, biomimicry, tissue regeneration, skin scaffold, fibroblasts

## Abstract

Skin autografts are in great demand due to injuries and disease, but there are challenges using live tissue sources, and synthetic tissue is still in its infancy. In this study, an electrocompaction method was applied to fabricate the densely packed and highly ordered collagen/sulfated xylorhamnoglycuronan (SXRGlu) scaffold which closely mimicked the major structure and components in natural skin tissue. The fabricated electrocompacted collagen/SXRGlu matrices (ECLCU) were characterized in terms of micromorphology, mechanical property, water uptake ability and degradability. The viability, proliferation and morphology of human dermal fibroblasts (HDFs) cells on the fabricated matrices were also evaluated. The results indicated that the electrocompaction process could promote HDFs proliferation and SXRGlu could improve the water uptake ability and matrices’ stability against collagenase degradation, and support fibroblast spreading on the ECLCU matrices. Therefore, all these results suggest that the electrocompacted collagen/SXRGlu scaffold is a potential candidate as a dermal substitute with enhanced biostability and biocompatibility.

## 1. Introduction

Skin is the largest organ of the human body and it acts as a physical barrier against the external environment. Compromised wound healing is a major issue in the treatment of massive skin lesions. Currently, widely used strategies to treat skin wounds include wound dressing, skin autografts and allografts [1]. However, these applications are limited by various disadvantages, such as low adhesion to lesions, donor shortage, and immune rejection [1]. Advances in tissue engineering have made the production of artificial skin possible. 

Skin tissue engineering alleviates the issue associated with donor shortage and prompts wound management via the use of bioactive cell-material complex scaffolds [2]. Currently, available tissue engineered skin substitutes include epidermal substitutes, dermal substitutes and epidermal-dermal substitutes [3]. While skin is the first type of tissue that has been successfully engineered for implementation into the clinical application, tissue-engineered skin still faces a number of challenges, limiting its wider application. These include the inability to mimic the composition and structure of natural skin, poor vascularization, scar formation, missing appendages and pigmentation and high manufacturing costs [4]. Typically, tissue-engineered skin involves the use of biodegradable materials to either induce the ingrowth of surrounding cells or to act as temporary supports for transplanted cells to attach, proliferate and differentiate to enable neo-tissue genesis. Thus, the role of scaffold material is crucial and this material should be carefully selected. Among the wide range of available biocompatible materials, including synthetic polymers and natural polymers, preferences are given to natural polymers such as collagen, elastin, and polysaccharides, because of their biocompatibility, biodegradability and presence of bonding sites with cells and bioactive molecules [5].

In skin tissue, collagen is the most abundant component in the extracellular matrix, providing integrity, rigidity and elasticity [6]. Collagen has thus been pursued in wound healing for several decades [3]. In particular, type I collagen is of key importance for cell-material interactions, as it contains crucial integrin-binding sequences including the arginine-glycine-aspartic acid (RGD) and glycine-phenylalanine-hydroxyproline-glycine-glutamic acid-arginine (GFOGER) sequence [7]. However, traditionally fabricated collagen hydrogels mainly contain randomly distributed fiber and cannot adequately emulate the complexity of natural ECM and skin mechanical properties. Collagen electrocompaction, which is a collagen densification method that takes advantage of the amphoteric nature of the collagen molecules to isoelectrically compact collagen molecules into densely packed and highly ordered bundles [8], is a promising method to fabricate collagen sheet that mimics the alignment, density and mechanical properties of collagen in skin tissues. 

In natural skin, apart from collagen, polysaccharides are also important native components with functions including maintaining moisture environment, promoting angiogenesis, offering binding sites for drugs or growth factors [5]. Recently, marine biomass has gained increasing attention as an abundant and sustainable source of polysaccharides. Among them, sulfated xylorhamnoglycuronan (SXRGlu), a sulphated polysaccharide extracted from the cell wall of specific green algae, is a promising candidate to explore. SXRGlu shares chemical similarities with natural-skin glycosaminoglycan polymers such as dermatan sulfate and hyaluronic acid [9], and from our previous lab trials with this species-specific extract, has shown a strong binding affinity to fibroblast cells and collagen, as well as organized structure and anti-inflammatory activity. Thus, the incorporation of SXRGlu is a strong candidate ingredient in electrochemically aligned collagen, as it may serve as suitable skin scaffold component substitutes for skin regeneration and wound healing. 

The aim of this project is to fabricate biomimicry dermal substitutes using electrochemically aligned collagen (ECL) matrix and SXRGlu for wound healing applications. SXRGlu was introduced to the ECL matrices by chemical crosslinking, using 1-ethyl-3-(3-dimethylaminopropyl) carbodiimide and *N*-hydroxysuccinimide (EDC–NHS). Physiochemical properties of the fabricated matrices including micromorphology, SXRGlu content and distribution, mechanical property, water uptake ability and degradability were evaluated. In addition, HDFs were cultured on the scaffolds to assess the cell viability, proliferation and morphology. 

## 2. Materials and Methods 

### 2.1. Synthesis of Electrocompacted Collagen Matrices

**Figure 1:**
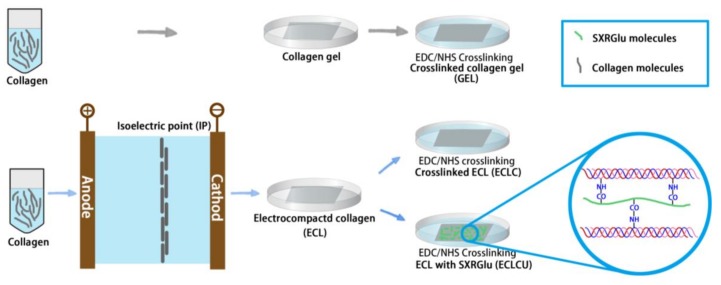
Schematic illustration of the preparation of electrocompacted collagen matrices (grey and green rods represent collagen and collagen/sulfated xylorhamnoglycuronan (SXRGlu) molecules respectively, colorful line in the blue circle represents collagen molecules). The colorful waves in the magnification represent electrocompacted collagen attached to SXRGlu (green).

The fabrication process of traditional collagen gel (GEL), electrocompacted and crosslinked collagen (ECLC) as well as SXRGlu-conjugated ECLC (ECLCU) matrices is illustrated in Figure 1. Type-I collagen powder (Kele Biotech Co., Chengdu, China) was dissolved in 0.5 M acetic acid at 6 mg/mL and dialyzed against ultrapure water at 4 °C for 12 h. Then the dialyzed collagen was loaded into a circular rubber washer (diameter = 1.2 cm and thickness = 2 mm) sandwiched between two oppositely charged electrodes. Upon application of an electric field of 6 volts for 15 min, a pH gradient was generated between the electrodes. Due to the amphoteric nature of the collagen molecules, the collagen molecules would be driven by the electrostatic repulsion from the electrodes and compacted along the isoelectric point thus forms a transparent “wet” collagen sheet (ECL). Then the freshly aligned collagen sheet was incubated in phosphate buffered saline (PBS) at 37 °C for 4 h for fibril formation. 

To enhance the mechanical property and stability of the collagen matrices, the ECL matrices were crosslinked using 20 mM EDC and 20 mM NHS in 50 mM 2-(*N*-morpholino) ethanesulfonic acid (MES) buffer for 4 h at room temperature [10]. The crosslinked ECL matrices (ECLC) were washed thoroughly with PBS to remove unreacted chemicals in the crosslinking solution.

Traditionally fabricated collagen gels were prepared as controls by mixing the dialyzed collagen solution (8 parts) with 10 × PBS (1 part) and adjusting the pH to 7.0–7.5 using 0.1 N NaOH (1 part). The mixture was then cast into molds and allowed to gel at 37 °C for 24 h followed by crosslinking using 20 mM EDC and 20 mM NHS in 50 mM MES buffer. Strips or circle membranes were then cut from these gels and processed as mentioned above for subsequent tests. An equal amount of collagen was used for fabrication of both compacted and uncompacted sheets.

### 2.2. SXRGlu Conjugation to ECL Matrices 

To mimic the more complex scaffold structure of skin matrices and investigate the functions of SXRGlu in wound healing applications, an SXRGlu rich extract, obtained from the cell wall of a DNA barcoded green macroalgae (PhycoTrix™, Venus Shell Systems, Bomaderry, Australia), was crosslinked to the ECL matrices by soaking the freeze-dried ECL matrices in 1% SXRGlu, 20 mM EDC and 20 mM NHS in 50 mM MES buffer for 4 h at room temperature. After that, the SXRGlu conjugated-ECL (ECLCU) were rinsed thoroughly with PBS, freeze dried and stored at 4 °C for further application.

To quantify the amount of SXRGlu in the ECLCU matrices, the ECLCU matrices were dissolved in 1 N HCl and incubated at 37 °C for 72 h. The dimethylmethylene blue (DMMB) assay [11] was used to quantify the amount of SXRGlu in the solution which indicated the SXRGlu amount that was crosslinked to ECLCU. To visualize the distribution of SXRGlu through the ECLCU, fluorescent labelling and confocal inspection were conducted. Fluoresceinamine labelled SXRGlu (Fluo-SXRGlu) was prepared by mixing 25 mM EDC, 25 mM sulfo-NHS, 1.25 mM fluoresceinamine (Sigma-Aldrich, Castle Hill, Australia) and 5% (*w*/*v*) SXRGlu into 0.1 M MES buffer (pH 6.0) and incubating under gentle stirring at room temperature for 24 h. Extra dyes were removed by dialyzing against distilled water for 7 days. Then the Fluo-SXRGlu was crosslinked to the ECL using the same procedures mentioned above.

### 2.3. SEM Inspection of the Electrocompacted Collagen Matrices

Scanning electron microscopy (SEM, JSM-7500FA Field Emission Scanning Electron Microscope) inspections were conducted on GEL, ECL, ECLC and ECLCU to investigate the effect of electrocompaction process on the microstructure of collagen. Samples used for SEM imaging were hexamethyldisilazane (HMDS, Sigma-Aldrich, Castle Hill, Australia) dehydrated. The fixing and dehydration process are as following [12]: freshly compacted collagen sample were immersed in PBS for 10 min then transferred to a fixing solution (2.5% glutaraldehyde in 0.2 M PBS, pH 7.0) for 0.5 h followed by washing with distilled water for 10 min. Samples were then immersed in graded ethanol series (50%, 75%, 90% and 100%) for 10 min at each concentration for dehydration and then in HMDS solution for 30 min and air dried in a fume hood at room temperature. Finally, samples were mounted on the SEM samples stub and coated with platinum (20 nm) using an Edwards sputter coater and then observed using SEM. 

### 2.4. Mechanical Property

Tensile mechanical properties of the traditional collagen GEL, ECLC and ECLCU were tested using a Shimadzu EZ-L universal mechanical tester at wet state with a 10 N load cell at a crosshead speed of 10 mm·min^−1^. Before testing, samples were washed with deionized water, dehydrated using a series of ethanol washes, and air dried. The dried bundles were glued onto transparency sheets of both ends using a UV curable glue (Dymax 425, Dymax Corporation, Torrington, CT, USA) and rehydrated with PBS [8]. The Young’s modulus was calculated by a linear regression fit of the strain-stress curve between the end of the toe region and the midpoint of the linear elastic region. For each reported value, three different samples were tested, and the results were averaged.

### 2.5. Swelling Ratio 

To evaluate the effect of electrocompaction and SXRGlu incorporation on the water up take behavior of collagen matrices, samples of 0.5 × 0.5 cm^2^ were weighed in the dry state and were recorded as (*W*_d_). Following this, samples were soaked in PBS for 24 h for complete hydration and then the wet weight (*W*_w_) was recorded after removing excess water on sample surface using Kimwipe. The swelling ratio of collagen matrices were calculated using the formula below [10]:

$$\mathrm{Swelling}\;\mathrm{ratio}\%=\left(\frac{W_{w}-W_{d}}{W_{d}}\right)\times 100$$

### 2.6. Degradability

The stability of electrocompacted collagen matrices was determined by assessing the resistance of the matrices to collagenase treatment. The collagen matrices were freeze-dried and the initial weight of the matrices prior to collagenase treatment was recorded (*W*_0_). Following this, the collagen matrices were immersed in the digest solution which contained 100 μg/mL (28 units) collagenase (type I, Sigma-Aldrich, Castle Hill, Australia) in PBS (pH 7.4) and incubated in water bath at 37 °C for desired time points. The digest solution was replaced every 2 h. At t = 2 and 4 h, the matrices were removed from the digest solution and freeze-dried. Then the residual mass at each time point was calculated.

### 2.7. Human Dermal Fibroblast Cell Viability, Proliferation and Morphology on Electrocompacted Collagen Matrices

Human dermal fibroblast (HDFs, Cell Applications, Inc., San Diego, CA, USA) cells at passage 8 were seeded onto the fabricated collagen scaffolds to assess the influence of electrocompaction and incorporation of SXRGlu on cell proliferation and morphology. The HDFs were cultured in Dulbecco’s Modified Eagle’s medium (DMEM) supplemented with 10% (*v*/*v*) fetal bovine serum, 100 U/mL penicillin, and 100 μg/mL streptomycin under 5% CO_2_ at 37 °C. Before cell seeding, scaffolds in 24 well plate were sterilized firstly by UV irradiation for 1 h, immersion in 70% ethanol for 3 h and washing for five times with PBS for 12 h. To saturate the scaffold with cell culture medium and facilitate cell attachment onto the collagen matrices, the sterilized scaffolds were incubated in 1 mL of culture medium at 37 °C overnight before cell seeding. 

The HDFs were then seeded at the density of 1.0 × 10^4^ cells/cm^2^ on the pre-processed scaffolds. Cell proliferation studies over 7 days were performed using the PrestoBlue™ (Life Technologies, Mulgrave, Australia) assay according to the manufacturer’s instruction. Briefly, at day 1, 3, 5 and 7, three HDFs-scaffold constructs were incubated with PrestoBlue™ mix for 1 h at 37 °C. Following this, for each sample, 100 μL aliquots of supernatant were transferred to a 96-well plate in triplicate and measured using a microplate reader (POLAR star Omega, BMG Labtech, Offenburg, Germany).

Live/dead assays were performed to inspect the HDFs cell viability on the scaffolds. Briefly, at each time point, HDFs-scaffold complexes were incubated with Calcein AM (5 μg/mL, Life Technologies, Mulgrave, Australia) at 37 °C for 10 min. Then, after a media change, propidium iodides (PI, 5 μg/mL, Life Technologies, Mulgrave, Australia) were added into the HDF-scaffold complexes and incubated for 5 min followed by a further media change. Images were acquired using a confocal microscope (Leica TSC SP5 II, Buffalo Grove, IL, USA) and the 3D projection tools in the Leica application suite X (LAS X) software (Leica, Buffalo Grove, IL, USA) were used for depth coding. 

Alexa Fluor 488-Phalloidin cell cytoskeleton staining (ThermoFisher Scientific, Waltham, MA, USA) were conducted to assess the cell morphology. To do this, collagen matrices with cells at day 7 were washed in PBS and fixed in 3.7% paraformaldehyde in PBS for 10 min at room temperature. Then, samples were washed with PBS and permeabilized with 0.1% Triton-X 100 in PBS for 5 min at room temperature. Samples were subsequently stained with Alexa Flour 488-Phalloidin (1:40 in PBS) for 1 h at room temperature, washed with PBS, and then incubated with 10 μg·mL^−1^ 4′,6-diamidino-2-phenylindole (DAPI, ThermoFisher Scientific, Waltham, MA, USA) for 10 min at RT to visualize the nuclei. Finally, samples were washed and imaged using a confocal microscope (Leica TSC SP5 II, Buffalo Grove, IL, USA).

## 3. Results and Discussion

### 3.1. SEM Inspection

**Figure 2:**
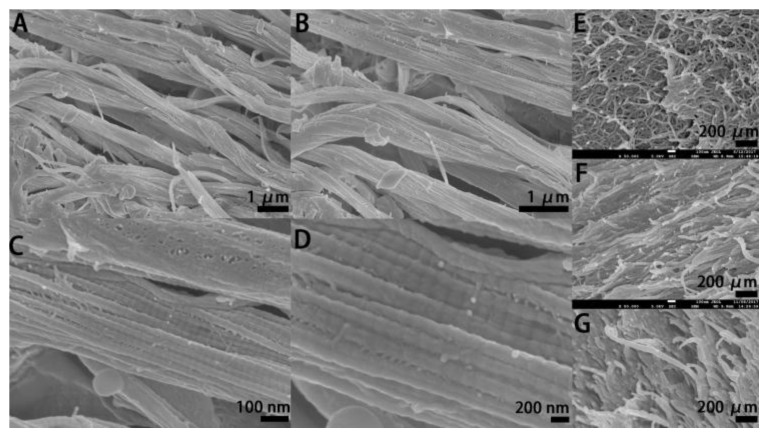
Representative SEM micrographs of the electrocompacted collagen matrices (ECL) before crosslinking at various magnifications (**A**–**D**), and crosslinked traditional collagen gel ((**E**), GEL), crosslinked ECL ((**F**), ECLC), crosslinked ECL incorporated with SXRGlu ((**G**), ECLCU).

SEM images of the electrocompacted collagen (Figure 2A–D) showed uniformly oriented collagen bundle which demonstrated successful electrocompaction whereas collagen gel without electrocompaction (Figure 2E) showed randomly aligned fibers with loosely packed pattern. Further, as can be seen in Figure 2D the D-banding pattern of collagen fibers are preserved after electrocompaction which confirmed that the electrocompaction process was not detrimental to the native microstructure of collagen molecules [8]. Besides, after EDC/NHS crosslinking (Figure 2F) and SXRGlu incorporation (Figure 2G), the highly ordered pattern of collagen fibers in ECL was retained. These results are consistent with previously reported works [10] and demonstrated that densely packed and highly ordered collagen matrices were successfully fabricated using the electrocompaction method.

Sherman et al. [13] reported the microscopic morphology of collagen fibers in rabbit skin which showed a well-organized, stacked and wavy structure of collagen fibers. Besides, rabbit skin exhibited highly orientated order and densely packed pattern of collagen fibrils within each collagen fiber as well as the D-banding pattern in collagen fibrils. Similar orientation, stacking, alignment and D-banding pattern were observed in our electrocompacted collagen matrices. Thus, the electrocompacted collagen matrices can preliminarily mimic the microstructure of collagen fibers in natural skin. Compared with other popular fabrication methods such as 3D printing [14], electrospinning [15], gas forming [16], phase separation [17], freeze-drying [18], collagen electrocompaction poses advantages. The whole electrocompaction process takes only 15 min and is comparatively rapid. In addition, this process is simple and highly cost-effective, especially compared with those requiring expensive specialized instruments, such as 3D printing. Furthermore, the whole process is organic solvent free while in many other methods, such as electrospinning, use of toxic solvents is indispensable. 

### 3.2. SXRGlu Content Quantification

**Figure 3:**
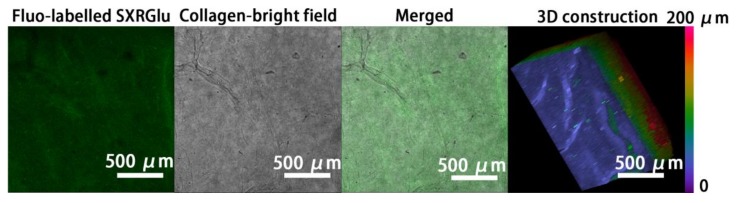
Confocal images of Fluo-labeled SXRGlu through the ECLCU matrices, and 3D reconstruction with the pseudo-color scale indicating depth coding of Fluo-labelled SXRGlu along the *Z*-axis (0–200 μm) (scale bar applied to all figures).

To mimic the typical matrix structure of natural skin, SXRGlu was conjugated to the ECL matrices. After SXRGlu conjugation, the final content of SXRGlu confirmed by the calibration curve in the ECLCU was 2.1 ± 0.2%. Confocal images of electrocompacted collagen conjugated with Fluo-labelled SXRGlu (Figure 3) confirmed uniform distribution of SXRGlu throughout the plane and depth of collagen. This structure preliminarily mimicked the components of the extracellular matrix in natural skin.

However, with the current EDC/NHS crosslinking method, the amount of SXRGlu that can be introduced into the ECLC matrices is limited, well below the composition of polysaccharides in native skin, among which collagen and polysaccharides account for 75% and 20% dry weight respectively. An alternative approach to improve SXRGlu loading is to develop SXRGlu derivatives that can carry both positive charge and negative charge, sharing the same isoelectric point with collagen [19]. In this way, SXRGlu and collagen can be co-electrocompacted at any ratio. Future work will be focused on polysaccharides modification, such as amination, and co-electrocompaction of polysaccharides and collagen to better mimic the ECM structure of natural skin.

### 3.3. Mechanical Property

**Figure 4:**
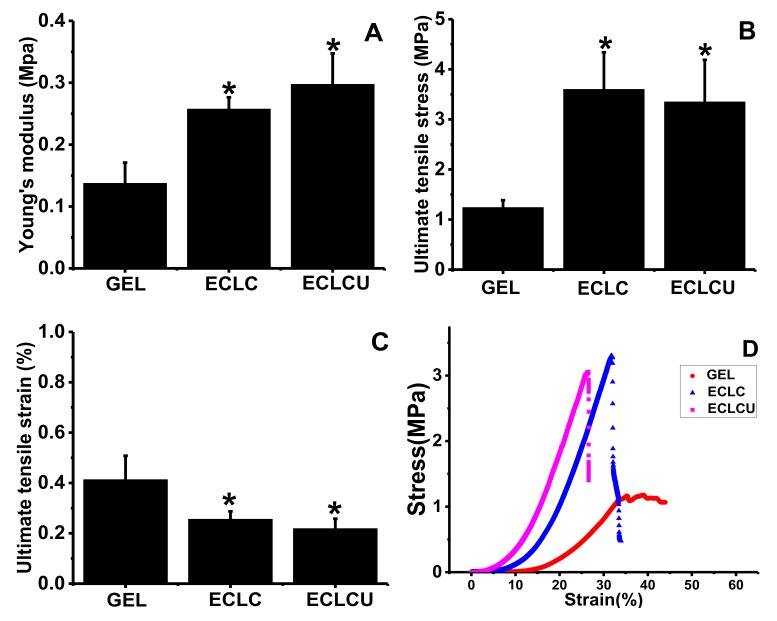
Tensile properties of GEL, ECLC and ECLCU. (**A**) Tensile modulus; (**B**) Ultimate tensile stress; (**C**) Ultimate tensile strain; (**D**) Stress-strain curve (* indicates significant difference compared with GEL at *p* < 0.05).

The Young’s modulus (Figure 4A), ultimate tensile stress (Figure 4B), ultimate tensile strain (Figure 4C) and strain-stress curves (Figure 4D) of the GEL, ECLC and ECLCU were obtained using tensile tests. The Young’s modulus of the ECLCU and ECLC was about 0.29 and 0.25 MPa respectively, which was significantly higher than that of collagen gel (0.13 MPa). The ECLCU had a 2-fold greater Young’s modulus and 2-fold greater ultimate tensile stress than the traditional collagen gel. In normal skin, the Young’s modulus measured from different skin sites is between 0.05 and 0.2 MPa [20]. Thus, the mechanical properties of the ECLC and ECLCU match natural skin very well. 

Yet, electrocompaction and crosslinking significantly decreased the extensibility of collagen matrices as shown by the ultimate tensile strain results. This might be due to the crosslinking and SXRGlu incorporation procedure reducing the mobility of collagen chains within the matrices, thus leading to a more rigid molecular structure which finally resulted in weaker elasticity [21]. This could be further improved by the incorporation of elastin component in the collagen scaffold. In skin tissues, fiber forming structural molecules, which include collagen, fibrin, fibronectin, elastin and fibrillin [22], define the rigidity and elasticity of skin tissue. Typically, the extracellular matrix of dermal skin comprises 70–80% collagen and 3–6% elastin [23]. Elastin fibers are accountable for the recoiling mechanism after deformation or stress [24]. Apart from its mechanical attribute, elastin-derived peptides have also shown to promote cell adhesion and proliferation [16], chemotacticity [25], and enhance protein kinase C activation ability [26] during the wound healing process. In subsequent studies, elastin will also be incorporated into the ECL system to increase the scaffold elasticity, thus better mimicking the mechanical property of natural skin ECM.

### 3.4. Swelling Ratio 

**Figure 5:**
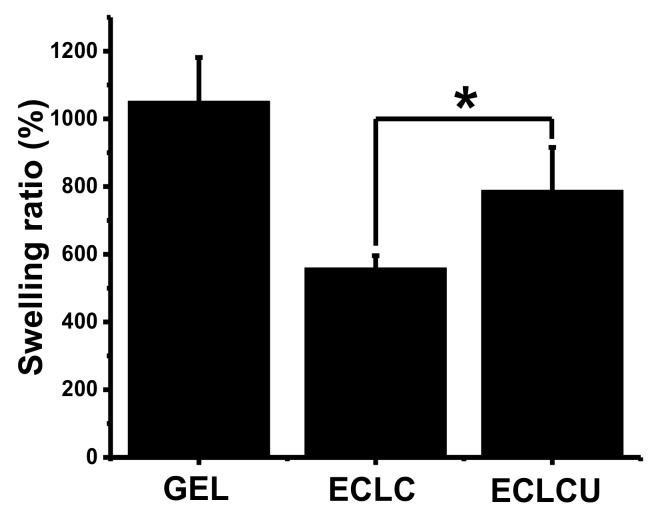
Swelling ratio of GEL, ECLC, and ECLCU (* indicates significant difference at *p* < 0.05).

The swelling ratio of GEL, ECLC and ECLCU were compared to evaluate the influence of the electrocompaction process and SXRGlu incorporation on the water uptake behavior of the matrices. As shown in Figure 5, the swelling ratio of GEL was around 1040%, while the electrocompacted collagen matrices (ECLC, ECLCU) exhibited reduced water uptake ability. This could be ascribed to their electrocompacted, densified structure of collagen fibers, which hindered the penetration of water molecules into the matrices. The incorporation of SXRGlu into the ECLCU matrices counteracted the effect of electrocompaction to a degree and increased the water uptake ability, which could be attributed to the hydrophilic nature of SXRGlu that is abundant in carboxyl, hydroxyl and sulfate groups. 

Apart from fiber-forming structural molecules, components in skin ECM can be divided into “matricellular proteins”, and “nonfiber-forming structural molecules” [27]. Matricellular proteins, such as thrombospondin-1 (TSP1), osteonectin, and osteopontin, mainly act as signaling molecules during wound healing stage and do not participate in the structural construction of ECM [28]. Non-fiber forming structural molecules are mainly glycosaminoglycans and proteoglycans such as hyaluronan, chondroitin sulfate, dermatan sulfate, decorin, lumican and versican [29]. They can fill in the gaps of interstitial space and function in buffering, hydration and force dispersion because of their negatively charged and hydrophilic nature. During the wound healing process, the maintenance of moisture near the wound site is a critical aspect of wound management, with demonstrated benefits, including accelerated angiogenesis, pain relief, prevention of tissue dehydration and cell death. Besides, if a scaffold is capable of swelling, it will be able to absorb bioactive molecules from neighboring tissue and thus promotes cell growth and function [30]. Therefore, incorporation of SXRGlu or other kinds of glycosaminoglycans (GAGs) into the collagen matrices can not only increase the water uptake ability but also better mimic the more structured scaffold environment of natural tissue, and as is required for prime cell behavior and functions. 

### 3.5. Degradability

**Figure 6:**
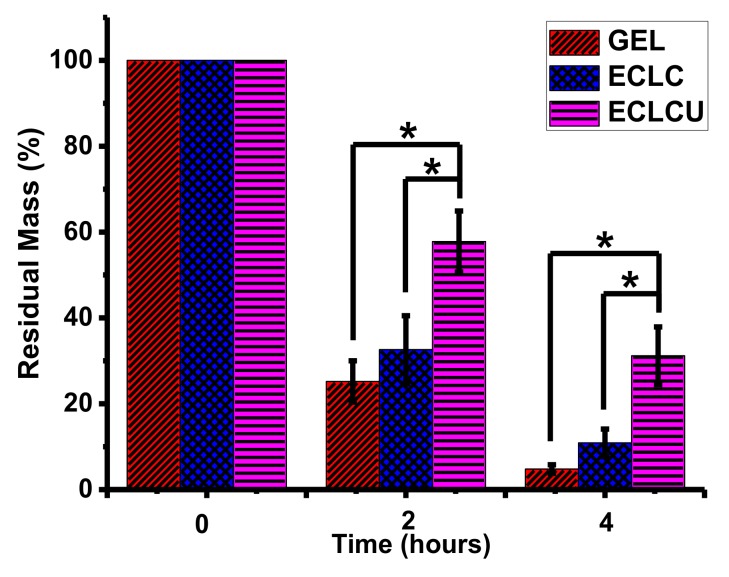
In vitro degradation of GEL, ECLC, and ECLCU matrices against collagenase (* indicates significant difference at *p* < 0.05).

Collagenase treatment to the collagen matrices was conducted to evaluate the influence of electrocompaction and SXRGlu inclusion on matrix stability. As shown in Figure 6, after 4 h, the residual mass was only 6% for GEL and 17% for ECLC, whereas the SXRGlu incorporated ECLCU remained highest at 36%. These data demonstrated that both the electrocompaction and incorporation of SXRGlu contributed to the improved resistance to collagenase mediated degradation. SXRGlu belongs to a group of algal cell wall extracts that are known for resistance to degradation from human endogenous enzymes [9]. Thus, the incorporation of SXRGlu into the ECL matrices may improve the stability of the collagen matrices in human physiologically relevant conditions.

### 3.6. Human Dermal Fibroblast Cell Viability, Proliferation and Morphology on Electrocompacted Collagen Matrices

**Figure 7:**
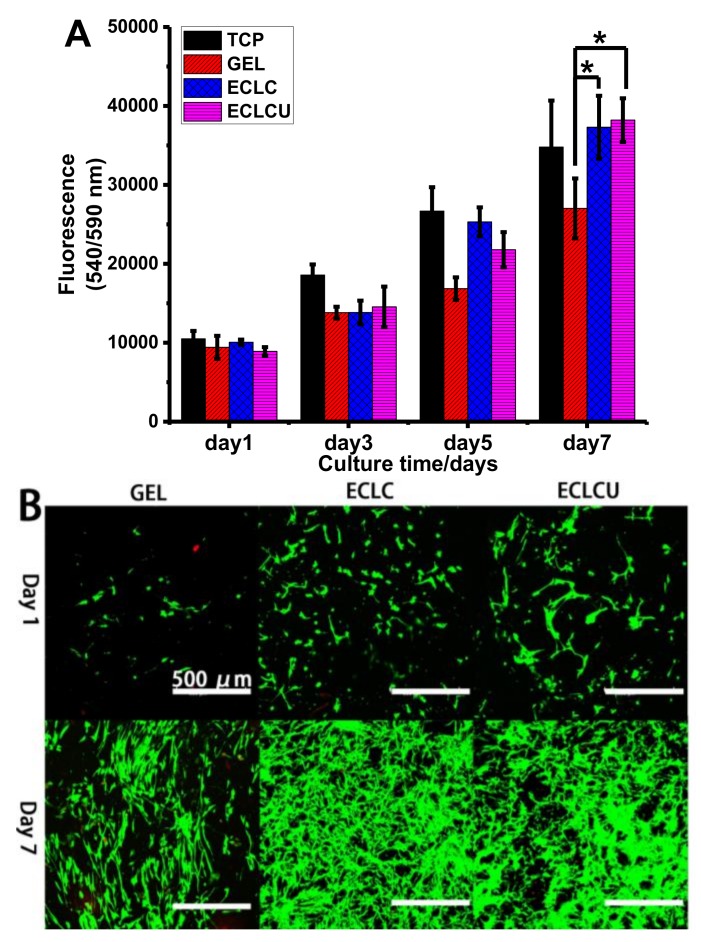
Survival and proliferation of human dermal fibroblasts (HDFs) on collagen matrix. (**A**) Proliferation (PrestoBlue™ cell viability indicator) of HDFs on tissue culture plate (TCP), GEL, ECLC and ECLCU matrices (* indicates statistical significance at *p* < 0.05 compared with cell numbers on GEL); (**B**) Live (stained by Calcein AM in green) and dead (stained by propidium iodide in red) HDFs at day 1 and day 7 (scale bar applied to all figures).

Cell studies were conducted to evaluate the viability, proliferation and cell morphology of HDFs cells cultured on the fabricated collagen matrices. The proliferation of HDFs on collagen matrices were assessed using PrestoBlue™ assay (Figure 7A). Quantitative measurement of cell viability marker PrestoBlue^®^ suggested that cell numbers on ECLCU and ECLC were higher than that on GEL on day 5 and day 7, which confirmed that the electrocompacted collagen matrices could promote the proliferation of fibroblasts. This is probably because the electrocompaction process increased the mechanical property of the matrix and this is in accordance with reports that fibroblasts responds to improved matrix stiffness by enhanced proliferation, collagen secretion, and smooth muscle α-actin (αSMA) stress fibers expression [31]. Especially, the proliferation of fibroblast was reported to be highly dependent on the stiffness of the matrices, and rigid ECM tend to induce the differentiation of fibroblast into contractile myofibroblast [31]. Over the 7-day culture, there is no significant difference in cell numbers between ECLC and ECLCU, which showed that the incorporation of SXRGlu did not inhibit the proliferation of HDFs. Live-dead assay on Figure 7B showed that nearly all cells were stained green with very few red cells implying that the HDFs cell viability was maintained on the electrocompacted collagen matrices. Compared to GEL, eletrocompacted matrices showed enhanced cell proliferation at day 7, which is consistent with the viability test using PrestoBlue™.

**Figure 8:**
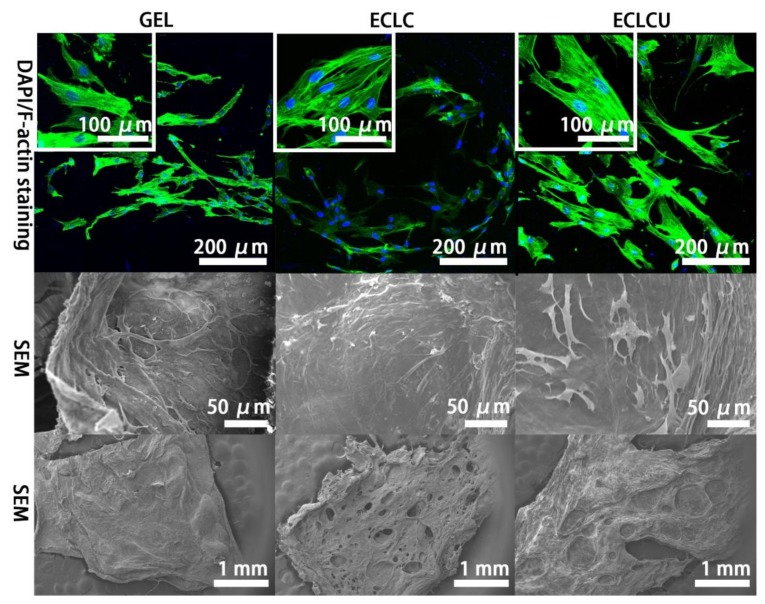
Representative F-actin filament staining with Alex-488 Phalloidin (staining F-actin in green)/DAPI (staining cell nucleus in blue) staining; SEM images of HDFs cultured on fabricated scaffold for 7 days; overall morphology of cell-material complexes after 7 days’ cell culture.

The morphology of HDFs on different matrices and the arrangement of the actin cytoskeleton were visualized by SEM inspection and fluorescent staining of cytoskeletal F-actin filaments after 7 days’ cell culture (Figure 8). F-actin morphology illustrated well-visible F-actin filament network on all these matrices suggesting HDFs attach and spread well on the GEL, ECLC and ECLCU matrices. Mean HDFs cell surface area calculated by Image J on GEL, ECLC and ECLCU are (960 ± 447), (1400 ± 278) and (2041 ± 214) μm^2^/cell (*n* = 100) respectively. HDFs on ECLCU resulted in most significantly increased F-actin area, indicative of most enlarged and spread out morphology of the HDFs. This might because the abundant rhamnose groups in SXRGlu could trigger skin fibroblast recognition and attachment to the ECLCU through the mediation of a unique rhamnose recognizing lectin-site on fibroblasts as receptor [32]. Besides, as shown on the SEM images of overall morphology of cell-material complexes on Figure 8, after supporting the HDFs for 7 days, the GEL matrices maintained the original flat shape while ECLC and ECLCU have shown more pores formed and the ECLCU seemed to exhibit better stability than ECLC. This might be due to cell numbers on the ECLC and ECLCU being significantly higher than on gel at day 5 and day 7 which might result in increased amount secretion of collagenase thus more severe degradation of the scaffold. As cell numbers on ECLC and ECLCU are similar, the less severe extent of degradation on the ECLCU compared with ECLC might be ascribed to the incorporation of SXRGlu. This is in accordance with our previous results that SXRGlu could improve the stability of the collagen matrices against collagenase degradation. 

Together, these results demonstrated firstly that electrocompaction of collagen improved the mechanical property of the collagen matrices and promoted HDFs proliferation; secondly, the incorporation of SXRGlu facilitated HDFs spreading and enlargement on the electrocompacted collagen matrices; also, the incorporation of SXRGlu increased the stability of the matrices against collagenase degradation. 

## 4. Conclusions

Herein we presented the fabrication and in vitro characterization of a biomimicking skin scaffold using collagen and SXRGlu. Electrocompacted collagen matrices were successfully fabricated. SEM inspection confirmed the presence of highly ordered, densely packed collagen fibers, mimicking the alignment and structure of collagen fibers in natural skin tissue more closely than the non-compacted matrix. Moreover, electrocompacted collagen matrices exhibited increased tensile strength and the mechanical properties of ECLC and ECLCU are comparable to that of natural skin. Incorporation of SXRGlu significantly improved the water uptake ability and stability of the electrocompacted collagen matrices. Cell proliferation studies showed the electrocompacted collagen matrices promoted the proliferation of HDFs, and SXRGlu facilitated HDFs spreading. Together, these results implied the potential of this SXRGlu incorporated electrocompacted collagen film as skin scaffold substitute.

In future work, polysaccharides including hyaluronic acid and SXRGlu will be aminated and co-electrocompacted with collagen and elastin to fully mimic the components and structure of natural skin ECM. Co-culture of keratinocytes and fibroblasts will be performed to do a reconstruction of a full thickness skin model.
